# The complete plastid genome sequence of *Chrysanthemum lucidum* (Asteraceae): an endemic species of Ulleung Island of Korea

**DOI:** 10.1080/23802359.2018.1463147

**Published:** 2018-04-23

**Authors:** Jung Sung Kim, Woong Lee, Jae-Hong Pak

**Affiliations:** a Department of Forest Science, Chungbuk National University, Cheongju, Republic of Korea;; b Research Institute for Dokdo and Ulleungdo Island, Kyungpook National University, Daegu, Republic of Korea;; c Department of Biology, Kyungpook National University, Daegu, Republic of Korea

**Keywords:** *Chrysanthemum lucidum*, endemic species, chloroplast genome, *rpoC1*; *ndhD*

## Abstract

The characteristic of complete chloroplast (cp) genome sequence of *Chrysanthemum lucidum,* one of famous insular plant and an endemic to Ulleung Island of Korea, was firstly introduced in the present study. It was 150,985 bp and contained a large single copy region (82,786 bp) and a small single copy region (18,281 bp) which were separated by two inverted repeat regions (24,959 bp). In total, 131 genes were identified and they were consisted of 76 coding genes, eight rRNA genes, and 36 tRNA genes. Comparing to the previously reported *Chrysanthemum indicum* and *C. x morifolium* cp genomes, we found complete inversion of SSC region in this taxa. *rpoC1* gene was pseudogenes due to 1 bp insertion of poly-A sequence in the 3′ of exon 2.

Genus *Chrysanthemum* L. (Asteraceae-Anthemideae) is comprised of about 40 species mainly distributed in East Asia and is well known for its commercial value as globally important cut flowers and pot plants (Liu et al. 2012). In Korea, three major groups, *C. boreale, C. indicum*, and *C. zawadskii* complex were recognized even their classification and circumscription have been still argued among the researchers (Kim et al. 2003). The plant evolution in the island is one of the top topics to botanists. Ulleung Island, which is small ocean island 150 km off the eastern coast of Korean peninsula, is famous for the high level of endemism in Korea (Oh et al. 2010). *C. lucidum* is one of the representative endemic species of Ulleung Island, used to be treated as variety or subspecies of *C. zawadskii* and the problem of its taxonomical phylogenetical position still remains.

We collected the plant material from the Ulleung Island of Korea, which is the important place for the plant evolution. The voucher was deposited at the Herbarium of Kyungpook National University (KNU). Complete chloroplast (cp) genome of *Chrysanthemum lucidum* (MH028788) was sequenced by HiSeq4000 of Illumina. Totally 47,670,990 paired-end reads (2 × 151 bp) were obtained and 3,626,189 reads were assembled to the reference cp genome of *C. indicum* (Xia et al. 2016) after reads end trimming with an error probability limit of 0.01. Then assembled reads were *de novo* assembled using the Geneious assembler. Using the assembled contigs, we conducted to align and repeat the procedure up to make a single contig. Complete cp genome was annotated using Geneious 10.2.3 (Kearse et al. 2012) with manual correction and tRNAScan-SE (Lowe and Eddy 1997) for tRNA gene.

It was a typical circular form with 150,985 bp in length and comprised a large single copy (LSC, 82,786 bp) region, a small single copy (SSC, 18,281 bp) region, and two inverted repeat (IR, 24,959 bp) regions. It was composed of 131 genes and they were identified 76 coding genes, eight rRNA genes, 36 tRNA genes, two pseudogenes. In the phylogenetic tree, the monophyly of the genus *Chrysanthemum* including *C. lucidum* was strongly supported (Figure 1).

**Figure 1.**
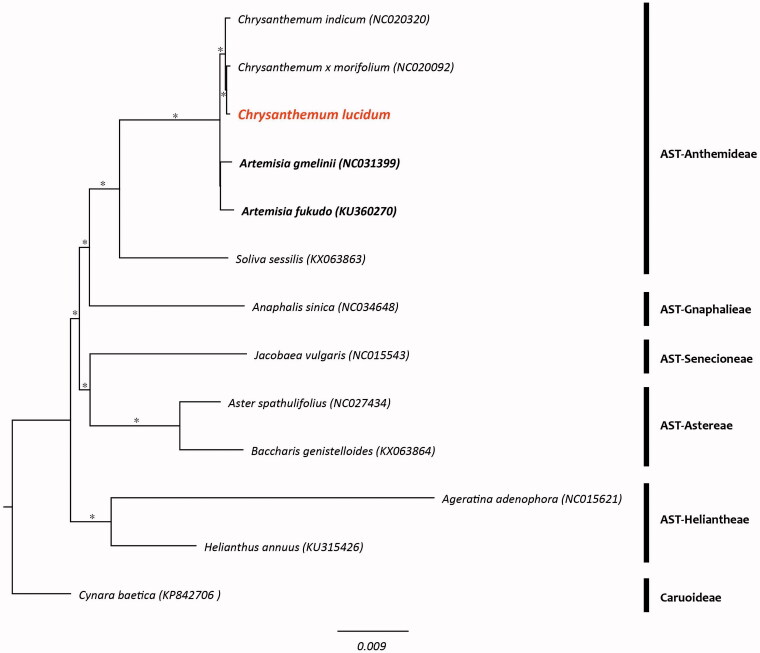
Phylogenetic tree of *Chrysanthemum lucidum* and related taxa using the complete chloroplast genome sequences. Asterisks indicate a branch which was supported by 100% bootstrap values. (AST: subfamily Asteroideae).

We found that indel in poly-A/T caused a pseudogenization in the genus. Both of *rpoC1* genes were pseudogenes due to 1bp insertion of poly-A sequence in the 3′ of exon2. Interestingly, complete inversion of SSC region, which has been reported from several members of Asteraceae family like *Artemisia*, was detected in *C. lucidum* cp genome.
